# Transcriptome-wide m6A methylation profiling of Wuhua yellow-feathered chicken ovary revealed regulatory pathways underlying sexual maturation and low egg-laying performance

**DOI:** 10.3389/fgene.2023.1284554

**Published:** 2023-10-20

**Authors:** Congjun Jia, Mengling Zhang, Xiaoyan Liu, Weilin Xu, Yanqing Xiong, Rihao Huang, Meidi Li, Mingna Li

**Affiliations:** ^1^ College of Agricultural Engineering, Guangdong Meizhou Vocational and Technical College, Meizhou, China; ^2^ Meizhou Engineering Research Center for Veterinary Medicine and Natural Medicine, Meizhou, China; ^3^ College of Animal Science and Technology, Gansu Agricultural University, Lanzhou, China

**Keywords:** Wuhua yellow-feathered chicken, ovary, m6A methylation, sexual maturation, reproduction

## Abstract

RNA N6-melthyladenosine (m6A) can play an important role in regulation of various biological processes. Chicken ovary development is closely related to egg laying performance, which is a process primarily controlled by complex gene regulations. In this study, transcriptome-wide m6A methylation of the Wuhua yellow-feathered chicken ovaries before and after sexual maturation was profiled to identify the potential molecular mechanisms underlying chicken ovary development. The results indicated that m6A levels of mRNAs were altered dramatically during sexual maturity. A total of 1,476 differential m6A peaks were found between these two stages with 662 significantly upregulated methylation peaks and 814 downregulated methylation peaks after sexual maturation. A positive correlation was observed between the m6A peaks and gene expression levels, indicating that m6A may play an important role in regulation of chicken ovary development. Functional enrichment analysis indicated that apoptosis related pathways could be the key molecular regulatory pathway underlying the poor reproductive performance of Wuhua yellow-feathered chicken. Overall, the various pathways and corresponding candidate genes identified here could be useful to facilitate molecular design breeding for improving egg production performance in Chinese local chicken breed, and it might also contribute to the genetic resource protection of valuable avian species.

## Introduction

The Wuhua yellow-feathered chicken (Supplementary Figure S1A) is an exclusive local breed native to Wuhua County in Meizhou City, Guangdong Province. It is known for its excellent meat quality and strong disease resistance ability (Weng et al., 2020). It is a small type of native chicken breed with relatively low growth and reproductive performance. Currently, it is primarily used as a meat-type breed and significant efforts have been made to improve its performance in meat production. The egg-laying performance, however, has been rarely studied. As a dwarf chicken breed, it possesses innate advantage of being an egg type strain, just like “Nongda No.3” grain-saving laying hens (Ning et al., 2013). Its small body size can help to conserve grain, thus alleviating competition for the grain between humans and livestock. It has been established that egg-laying performance is not only an economic trait, but also can serve as a very important reproductive characteristic. However, low reproductive performance has significantly limited both industrial development and genetic resource protection of the Wuhua yellow-feathered chicken. Therefore, there is an urgent need to improve the egg-laying performance of the Wuhua yellow-feathered chicken.

Egg-laying performance can be strongly associated with the chicken follicle development, which is a complex process controlled by the gonadal axis of the reproductive endocrine system (Johnson, 2012). Before attaining sexual maturity chicken ovary contains numerous quiescent primordial follicles. At the onset of laying, however, follicles with different development stages develop in the sexually mature ovary at the same time. Interestingly, a previous study has indicated that a sexually mature ovary in hen contains approximately 12,000 oocytes, but only a few hundred oocytes were selected to reach maturity and ovulate (Onagbesan et al., 2009). During the egg-laying period, one single small yellow-feathered follicle (SYF) was chosen from the cohort of SYFs to develop into a hierarchal follicle every single day, which constitutes a complicated process termed as the follicle selection (Johnson and Woods, 2009). The selected follicles then grow fast and can eventually ovulate in a few days. Thus, the successful follicle selection is the fundament of egg production and reproductive performance. The development of chicken ovary follicle is mainly regulated by multiple genes related to the various biological processes such as steroid hormones biosynthesis, granulosa cell proliferation and differentiation (Woods and Johnson, 2005). In addition, a number of important epigenetic events, such as DNA methylation and RNA modifications, have been reported to be involved in regulation of chicken follicle development (Zhu et al., 2015; Fan et al., 2019).

m6A is the most abundant type of posttranscriptional RNA modification found in eukaryotic species (Dunin-Horkawicz, 2006). m6A is defined as the methylation of adenosine 6 positions in mRNAs and some non-coding RNAs. It is primarily controlled by three different regulators namely, methyltransferases, demethylases, and recognition factors. These three regulators are also known as “writers”, “erasers”, and “readers” of the m6A. As a kind of important epigenetics modifications, it has been found to be associated with various physiological processes including modulation of various reproductive traits in mammals. For instance, a comprehensive review article elegantly summarized the role of m6A on the female reproductive biology and pathophysiology in recent years (Huang and Chen, 2023). In the review, the authors have discussed all aspects of the physiological function of m6A in female reproductive system and related diseases. Interestingly, in yak, m6A was identified as the key epigenetic modification involved in the sexual maturity process of male yak (Wang et al., 2021) and in the development of follicles in the female yak (Guo et al., 2022). In chicken, there is only one study performed by using the commercial chicken breed to investigate the possible role of m6A in the follicle development so far (Fan et al., 2019). The function of m6A on the development of chicken ovary, especially in Chinese local chicken breed, remains largely unclear.

Thus, to better understand the dynamic changes and the functional relevance of m6A in chicken ovary during sexually maturity, we have profiled the transcriptome-wide m6A methylation in Wuhua yellow-feathered chicken ovary. The results of this study may provide important insights for understanding the underlying molecular mechanisms related to the sexual maturation in Chinese indigenous chicken breeds. The findings may also be important for genetic improvement of egg laying performance.

## Materials and methods

### Ethics statement

All animal experiments were approved by the Animal Ethics Committee of Guangdong Meizhou Vocational and Technical College (GDMZVTC-2022-001). All animal procedures were performed in strict accordance with the guidelines proposed by the Ministry of Agriculture and Rural Affairs of the People’s Republic of China.

### Animals and sample collection

Six healthy Wuhua yellow-feathered chickens, used for sample collection were originated from Wuhua yellow-feathered chicken reservation farm, Wuhua Country, Meizhou, Guangdong province of the People’s Republic of China. Among these, three hens were 14 week old (before laying period, named group BLP) and three were 35week old (during laying peak period, named group LPP). The whole ovary was collected from each animal after euthanizing within 10 min (Supplementary Figure S1B). The yolk in the follicles was carefully removed by phosphate buffered saline. After collection, the tissue samples were stored in liquid nitrogen for further RNA isolation.

### RNA isolation and construction of library for MeRIP-Seq and RNA-seq

Total RNA was isolated from each ovary sample using the TRIzol reagent (Invitrogen, Carlsbad, CA, United States) according to the manufacturer’s instructions. Both the concentration and purity of the RNA were measured using the NanoDrop 2000 (NanoDrop, Wilmington, DE, United States).

The mRNA was first enriched from the total RNA using the Dynabeads Oligo (dT) and then fragmented. The fragmented RNAs were then divided into two distinct parts, one for isolation of m6A enriched mRNA and the other used for RNA-seq as the input background. The former part of the fragmented RNAs was incubated with the m6A-Dynabeads at room temperature for 1 h to allow them to bind to the beads. Eluted m6A-containing fragments (IP) and untreated input control fragments (the latter part) were then concentrated to generate the final cDNA library, respectively. The libraries were thereafter qualified and absolutely quantified using an Agilent Bioanalyzer 2100 (Agilent Technologies, CA, United States). The prepared libraries were then sequenced on an Illumina Hiseq X Ten.

### Analysis of sequencing data

The raw reads, including data from MeRIP-seq and RNA-seq, were thereafter processed using the Trimmomatic (v 0.39) (Bolger et al., 2014) software to obtain the clean reads by removing the reads with adapter or ploy N as well as the reads with relatively low quality (Q < 15, read_length <50 bp). The clean reads were then aligned to the chicken reference genome GRCg7b (https://www.ncbi.nlm.nih.gov/datasets/genome/GCF_016699485.2/) by using the HISAT2 (v 2.1.0) (Kim et al., 2015) with default parameters. Finally, only unique reads with high mapping quality were retained for further analysis.

### MeRIP-seq

To access the quality of methylated RNA immunoprecipitation sequencing data, R package Guitar (Cui et al., 2016) was adopted.

After all the quality control procedures mentioned above were performed, MeTDiff (v 1.1.0) (Cui et al., 2018) was used to call the peaks of m6A modification in each group with the input data as the control (*p*-value < = 0.05, fold change > = 1.5). When calling m6A peaks, all the parameters were set in default (except the option FRAGMENT_LENGTH = 200). To investigate the possible difference in m6A modification between group BLP and group LPP, the MeTDiff was used again (FRAGMENT_LENGTH = 200). The annotation of the identified m6A peaks in each group and differential m6A peaks between groups was carried out by ChIPseeker (v 1.12.1) (Yu et al., 2015).

The sequence motifs of m6A sites were detected using DREME (v 5.5.2) (Bailey, 2011). The functions of the m6A modification genes were investigated using GO and KEGG analysis. GO analysis was performed using the tools offered by the Gene Ontology Consortium (http://geneontology.org/). KEGG analysis was conducted using tools offered by the Kyoto Encyclopedia of Genes and Genomes (https://www.genome.jp/kegg/).

### RNA-seq (input data)

The input data were used determine the levels of gene expression and used as the background in m6A peak calling. After the quality control, the HTSeq (v 0.9.1) (Anders et al., 2015) software was employed to obtain the number of reads which were located in each protein coding gene. The number of reads captured was then normalized using the algorithm called Fragments Per Kilobase of Transcript Per Million Fragments Mapped (FPKM) (Roberts et al., 2011) by Cufflinks software (v 2.2.1) (Trapnell et al., 2012). The differential expression genes were thereafter identified using DESeq2 (v 1.18.0) (Anders et al., 2012) with the criteria fold change in FPKM >2.0 or <0.5, and *p*-value≤0.05. The function of the differential expression gene was investigated using GO and KEGG analysis.

### Combination analysis of MeRIP-seq and RNA-seq

To reveal the potential functions of dynamic m6A modification in regulating mRNA function during the development process of Wuhua chicken ovary, we examined the correlation between the gene expression levels and the abundance of m6A peaks based on the calculated fold changes. Generally, the m6A peaks with a Log2 fold change >0.5 or < −0.5, *p*-value <0.01, and the corresponding genes with a Log2 fold change >0.5 or < −0.5, *p*-value <0.01 were considered to be significant in the combination analysis.

## Results

### Morphological characteristics of chicken ovary

**FIGURE 1:**
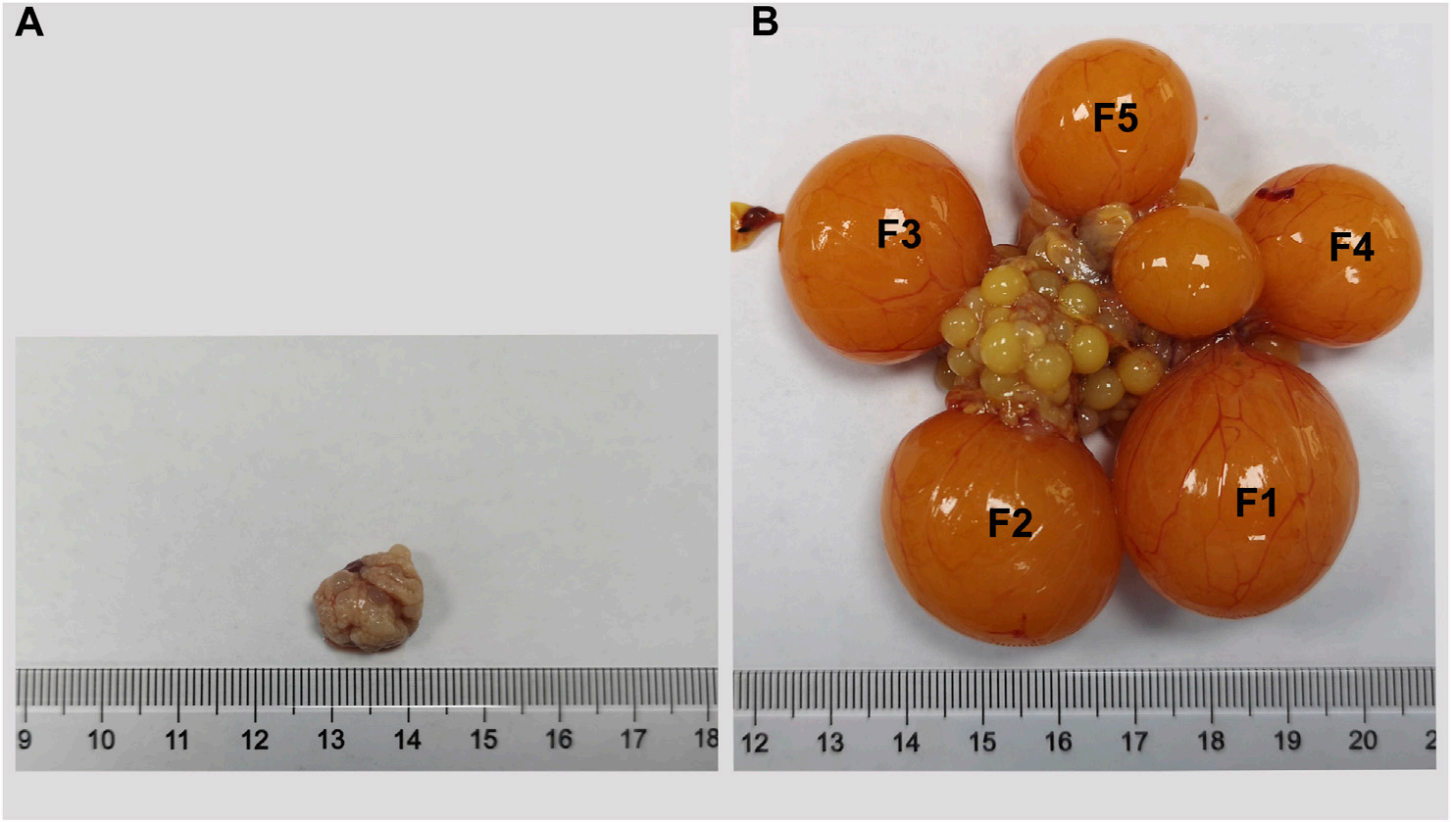
The ovary of Wuhua yellow-feathered chicken before **(A)** and after **(B)** sexual maturation.

To decipher the transcriptome-wide m6A methylation profiles during the process of Wuhua yellow-feathered chicken ovary development, we collected the ovaries at two contrasting stages (BLP vs. LPP, Figure 1) in triplicates for MeRIP-seq. Based on the picture of ovaries sampled at two different stages, we observe the distinct differences in both shape and size of ovaries. In generally, ovaries before the sexual maturity are small, and the follicles present on it are found in the primordial follicle stage. After the sexual maturity, however, the size of ovary is times bigger than that before the maturation. There were follicles of different sizes observed in various development stages in one ovary, which can be roughly divided into two parts, namely, pre-hierarchical follicles and hierarchical follicles (F1-F5).

### General features of m6A methylation in chicken ovary before and after sexual maturation

The number of raw reads getting from the MeRIP-seq of each sample ranged from 52.17 M to 62.99 M. After the quality control, we obtained 51.99 M–55.83 M clean reads for each sample. For RNA-seq, the number of raw reads varied from 48.19 M to 76.88 M for each sample, and the clean reads number was 47.97 M–54.93 M for each sample. The detailed information of the sequencing data has been listed in the Supplementary Table S1. The mapping statistics of the clean reads has been reported in Supplementary Table S2, Supplementary Figure S2A, and only the unique high-quality reads were used for the following analysis.

We identified 24,830 and 25,293 m6A peaks in chicken ovary of BLP and LPP group, respectively. The statistical analysis of the identified peaks has been displayed in additional files (Supplementary Table S3, Supplementary Figures S2B–D).

**FIGURE 2:**
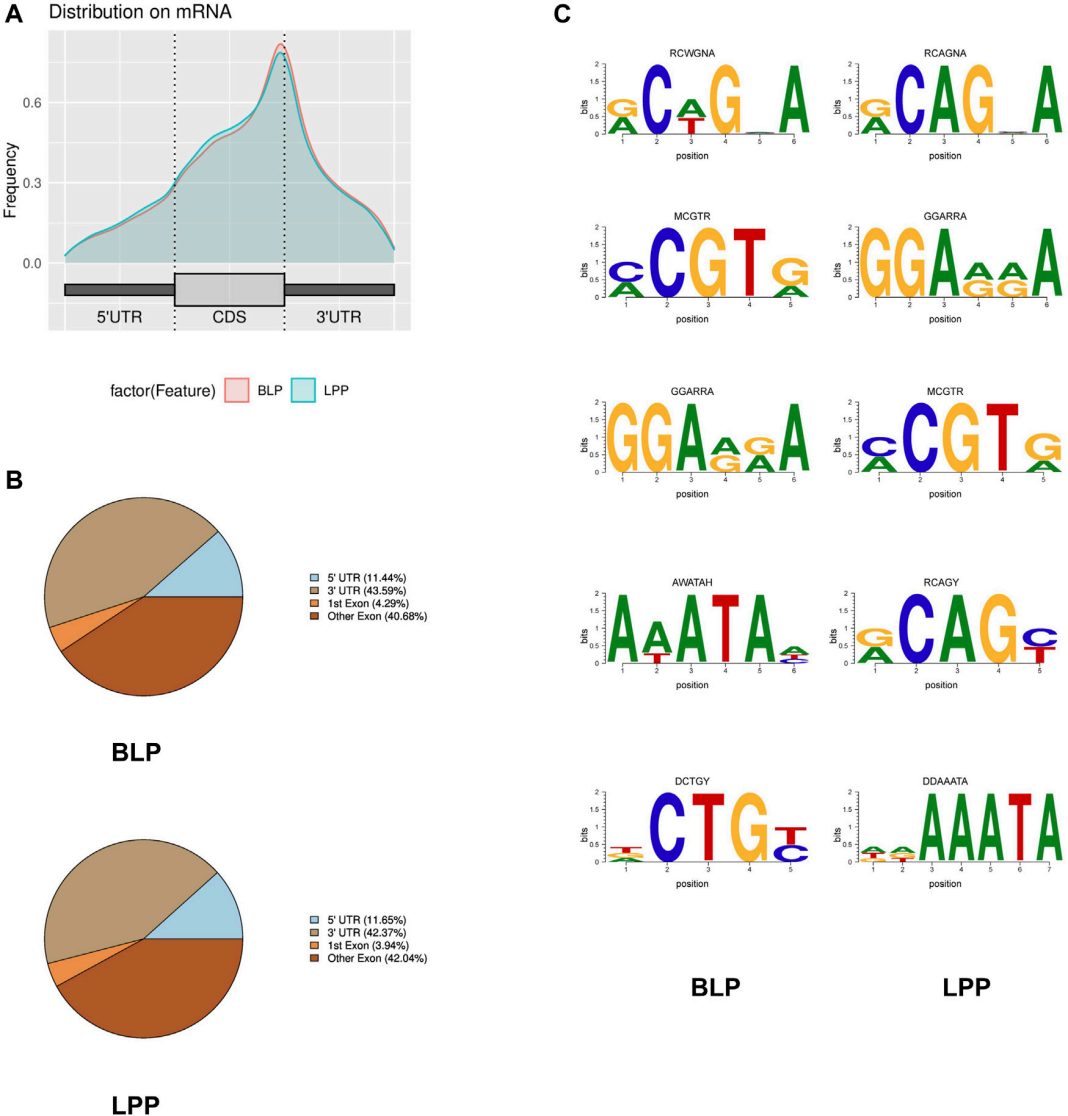
Overview of the m6A methylation profile in Wuhua yellow-feathered chicken ovary. **(A)** Distribution of m6A peaks along transcripts. **(B)** Proportion of m6A peaks fallen along transcripts. **(C)** The top motifs enriched across m6A peaks identified from BLP and LPP.

To further investigate the distribution of m6A peaks across the transcript, we annotated the peaks that were identified. The results demonstrated that the peaks were markedly enriched in the exon region (CDS) and stop codon in both BLP and LPP groups (Figures 2A, B).

To determine the motifs present in m6A peaks, we scanned each peak, and the top 5 sequence motifs in BLP and LPP have been listed in Figure 2C. We observed that both MCGTR (M = A or C; R = A or G) and GGARRA (R = A or G) were significantly enriched in m6A peak sites in chicken ovary.

### Differential m6A methylation analysis

**FIGURE 3:**
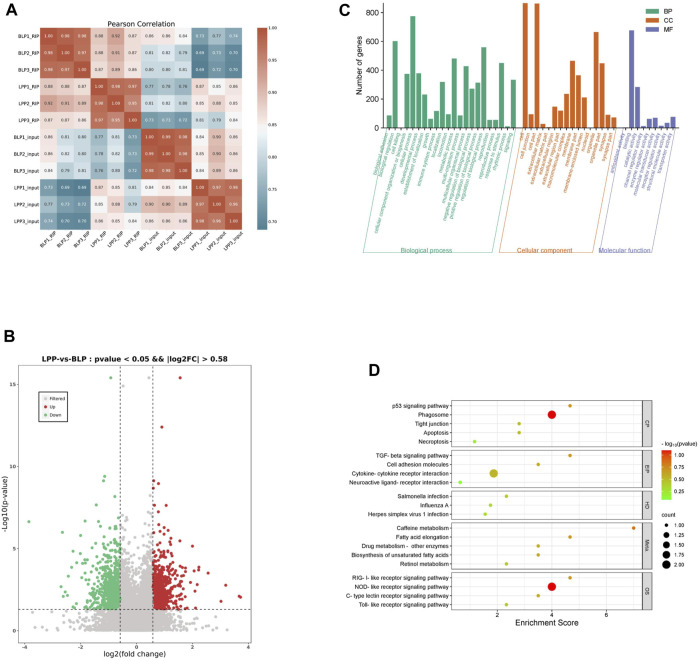
Analysis of differential m6A methylation in Wuhua yellow-feathered chicken ovary. **(A)** Heatmap of the sample correlation matrix based on sequence data. **(B)** The volcano of differential methylation peaks (DMPs). **(C)** GO analysis for genes with DMPs. **(D)** KEGG pathway analysis of genes with DMPs.

The m6A results of biological replicates within each group exhibited high concordance using the Pearson correlation coefficient (Figure 3A), which suggested that the samples we used is the present study were of good quality and suitable for analysis. To further detect the dynamic changes of the m6A methylation in two distinct physical phases, we assessed the differentially methylated m6A peaks (DMPs). Interestingly, differential peak analysis revealed that 1,476 DMPs between these two groups (Supplementary Table S4), which could be potentially associated with the development of chicken ovary. As it listed in Table 1, the total length of the DMPs were 3,583,532 bp with an average length of 2,427.87 bp, which represents about 0.35 percent of the chicken genome. Among the various DMPs identified, it was found that compared to BLP, the number of significantly upregulated methylation peaks and downregulated methylation peaks was 662 and 814 in LPP group, respectively (Figure 3B). The number of genes corresponding to the up DMPs and down DMPs was 580 and 763, respectively.

**TABLE 1:** Statistics of the differential m6A peaks identified between BLP and LPP.

Sample name	LPP-vs-BLP
Number of Peaks	1,476
Total Length of Peaks (bp)	3,583,532
Average Length of Peaks (bp)	2427.87
Median Length of Peaks (bp)	300
Percentage of Genome (%)	0.34

To better understand the possible functional consequences of m6A methylation, we performed a functional enrichment analysis on the various genes containing DMPs. The significantly enriched GO terms have been listed in Supplementary Table S5; Figure 3C. The significant terms in the biological process category were related to reproduction, reproductive process, rhythmic process, growth, multicellular organismal process etc. The KEGG analysis revealed that the genes associated with DMPs were mainly enriched in pathways like Retinol metabolism, p53 signaling pathway, Apoptosis, Necroptosis, TGF-beta signaling pathway, Cytokine-cytokine receptor interaction, Toll-like receptor signaling pathway, etc (Supplementary Table S6; Figure 3D).

### Differentially expressed genes (DEGs) analysis

**FIGURE 4:**
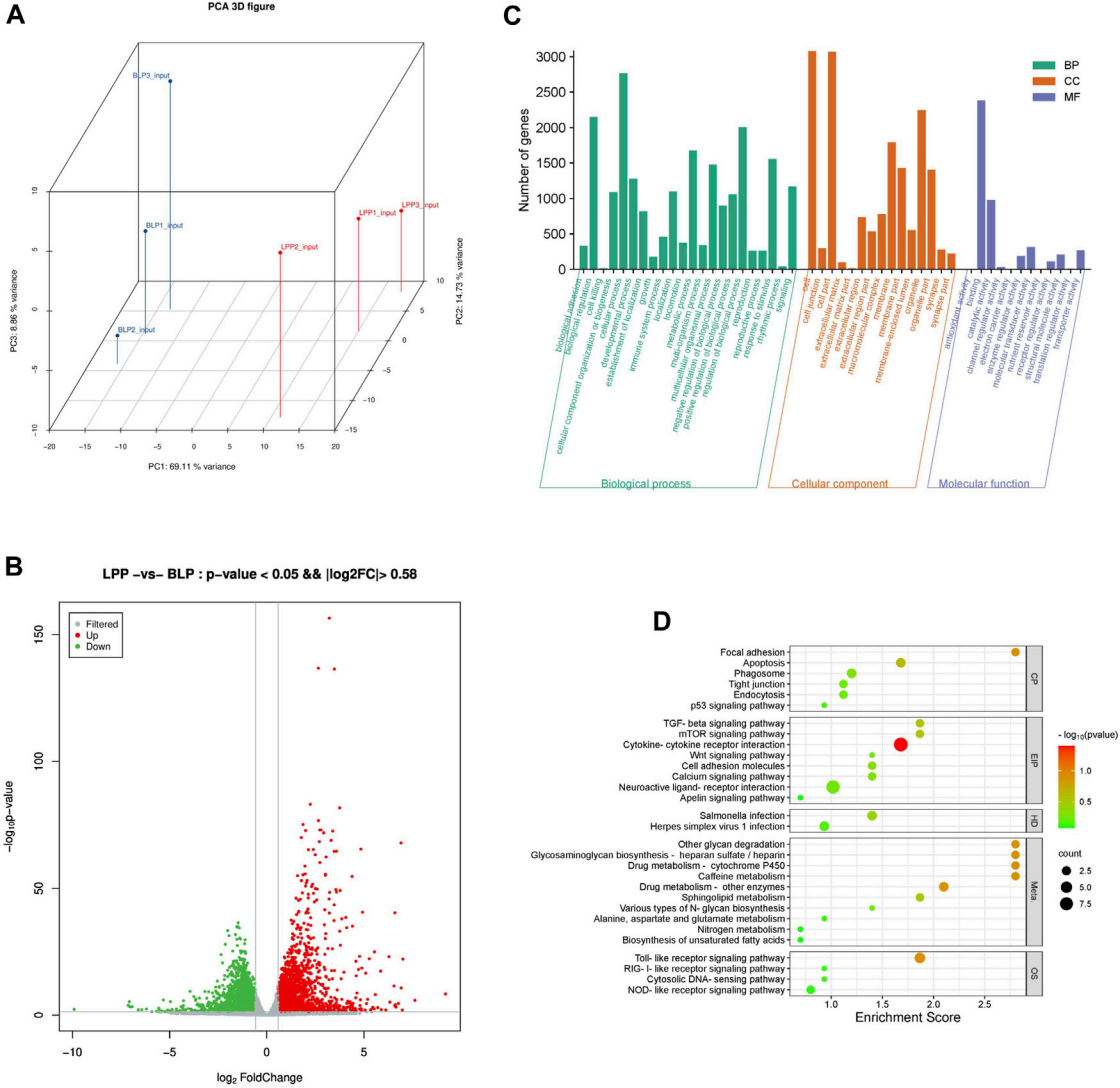
Analysis of differentially expressed genes (DEGs) in Wuhua yellow-feathered chicken ovary. **(A)** PCA of the samples based on sequence data. **(B)** The volcano of DEGs. **(C)** GO analysis for DEGs. **(D)** KEGG pathway analysis of DEGs.

A principal component analysis (PCA) based on the RNA-seq data (Figure 4A) displayed a high concordance within each group and a clear separation between the two groups, which indicated that the further analysis could be quite reliable. Through analyzing the input data, we found that a total of 4,354 genes were differentially expressed between these two groups. Moreover, in comparison to BLP group, there were 2,282 upregulated DEGs and 2,073 downregulated DEGs found in the LPP group (Supplementary Table S7; Figure 4B).

GO analysis demonstrated that the DEGs were mainly related to reproductive process, reproduction, developmental process, metabolic process and growth, which were vital important for the development of follicles (Figure 4C). Interestingly, KEGG pathways (Figure 4D) found to be significantly enriched were follicles development related pathways, such as mTOR signaling pathway, TGF-beta signaling pathway, Wnt signaling pathway, MAPK signaling pathway, VEGF signaling pathway as well as some cell growth and death related pathways such as Apoptosis and p53 signaling pathway.

### Correlation analysis of m6A methylation and DEGs

**FIGURE 5:**
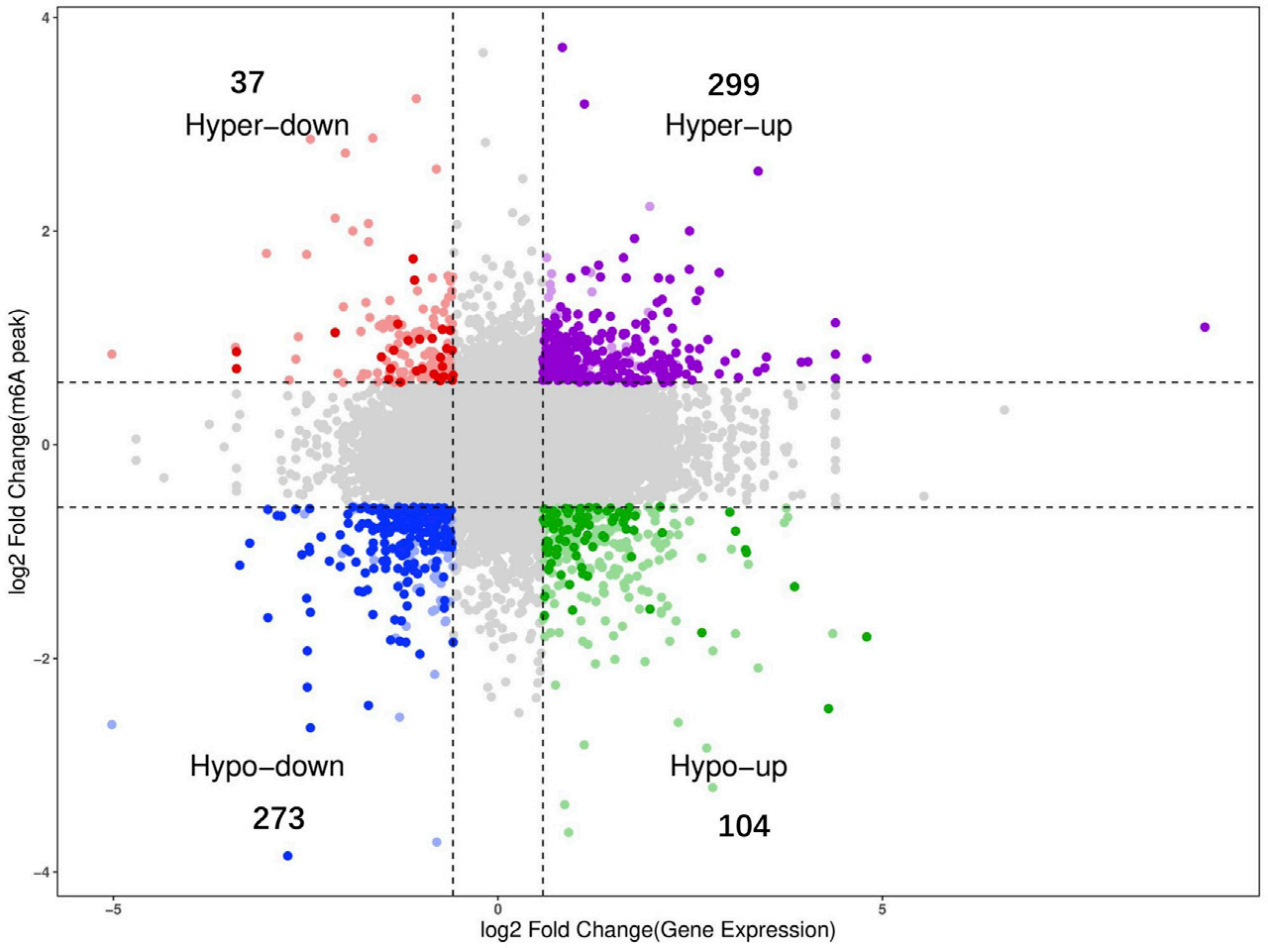
Conjoint analysis m6A-seq and RNA-seq data (gray dots indicating genes with no significant differences; colored dots indicating genes with significant differences).

To determine the potential functions of dynamic m6A modification in regulating mRNA function during the development of Wuhua chicken ovary, we examined the possible correlation between the gene expression levels and the abundance of m6A peaks based on the calculated fold changes. The results of the correlation analysis revealed a positive correlation between the global RNA methylation and gene expression levels (Figure 5). Overall, we found that 713 mRNAs were significant both in m6A levels and gene expression levels (Supplementary Table S8). Among them, 299 mRNAs were “hyper-up” type, which means that compared to BLP group, both m6A and the gene expression levels were upregulated. The number of “hypo-down” type mRNAs was 273, which indicated that both levels were downregulated. The number of “hyper-down” mRNAs and “hypo-up” mRNAs were found to be 37 and 104, respectively.

## Discussion

Egg laying performance is an important trait in poultry industry, as it is not only an economic trait affecting the egg production in hen industry but also has a reproductive value which might restrict the development of meat chicken industry. Ovary development is an important biological process that can primarily determine the laying performance in hens. However, the molecular regulation mechanisms underlying this development process remains largely unknown, especially for Chinese local chicken breeds which usually exhibit relatively poor egg laying performance. Moreover, previous studies related to chicken ovary development or follicle selection have suggested that the molecular mechanisms underlying this process is relatively complex, and epigenetic modifications such as m6A methylation play a critical role in regulating this physiological process (Fan et al., 2019). In this study, we have investigated the dynamic changes of transcriptome-wide m6A methylation of the Wuhua yellow-feathered chicken ovary before and after the sexual maturation stage. To our best knowledge, this is the first study to decipher the possible role of m6A modification in ovary development of Chinese indigenous chicken breed. Our data indicated that m6A levels of mRNAs changed significantly, thus indicating that m6A might play an important role in the process of ovary development. Further analysis revealed that apoptosis related pathways could be the key molecular regulatory cascades underlying the poor reproductive performance of Wuhua yellow-feathered chicken in comparison to other commercial laying hens breed such as Hy-line Brown chicken. However, the specific methylase and the regulatory mechanisms are still unclear and hence more further studies are required.

In the present study, we have profiled the transcriptome-wide m6A distribution in Wuhua yellow-feathered chicken ovary tissues at different stages of development. The results demonstrated that the transcripts in ovary were extensively methylated, which indicated that m6A may be involved in the development of the ovary. Similar to other studies (Fan et al., 2019; Wang et al., 2021; Guo et al., 2022; Li et al., 2022), we found that m6A peak site was remarkedly enriched in the CDS and 3′UTR, especially in the vicinity of the stop codons. Accumulating evidences suggest that the methylation site on a transcript may affecting the fate of the transcript (Dominissini et al., 2012). It has been hypothesized that the methylation on the internal exons might control the splicing of the transcript and the methylation near the stop codon could influence the translation of the transcript. Taken these together, we speculate that m6A could play a major role in regulating the development of Wuhua chicken ovary. To further investigate the potential role of m6A in the process, we combined the gene expression and m6A levels together to analysis the potential relationship between them. Interestingly, a positive relationship was observed, which indicated the m6A might regulate the mRNA levels and thus influence the process of follicle development.

The chicken ovary development consists of many complex biological processes, including follicle selection, follicular somatic cells transformations, and angiogenesis (Matzuk, 2000; Fortune et al., 2001; Hillier, 2001). Many studies focusing on the molecular mechanisms of follicle selection were conducted, and some potential pathways controlling follicle selection were found. In the present study, we have found that mTOR signaling pathway, TGF-beta signaling pathway, Wnt signaling pathway, MAPK signaling pathway, and VEGF signaling pathway were enriched by the differential expression genes, indicating these pathways may be important in the development of the ovary. Consistent with our study, TGF-beta signaling, Wnt signaling and Steroid hormone biosynthesis pathways have been reported to be significantly enriched in follicle selection in the hybrids of Huiyang Bearded and White Leghorn chickens (Nie et al., 2022). After the follicle selection, the selected follicle then can rapidly develop and form a mature yolk. The growth in size is accomplished by concurrent increase in the vasculature and blood flow, which can enable the follicle to accumulate large amounts of nutrients to form the lipid-rich mature yolk (Johnson, 2015; Nie et al., 2022). Interestingly, VEGF signaling pathway together with Wnt signaling pathway, significantly enriched in the present study, was identified to play an important role in the angiogenesis in the ovary during sexual maturity (Johnson, 2015).

As a kind of RNA modification, m6A is a common posttranscriptional regulation mode of gene expression. It can affect the fate of the RNA, such as by affecting the stability of RNA to determine whether the RNA is degraded or not, and then affect the corresponding biological processes (Dominissini et al., 2012). Interestingly, a positive relationship between m6A methylation and gene expression was observed in this study, which indicating m6A can regulate the development of ovary through affecting key genes associated with this process. Retinoids, consisting of retinol and its derivatives, can play a vital role in the development and maintenance of the normal physiological functions of the ovary (Liu et al., 2018b). A number of previous studies have revealed that retinoic acid (RA), one derivatives of the retinol, could substantially promote GC differentiation and oocytes maturity and ovulation in female mammalian (Ikeda et al., 2005; Tahaei et al., 2011; Kawai et al., 2016). The retinol level in the fluid of the follicles varies according to the different development stages with highest concentration found in dominant follicles relative to small follicles (Brown et al., 2003). Interestingly, the “Retinol metabolism” pathway was observed to be enriched by the DMPs in the present study. The corresponding gene within this pathway identified was *CYP2W1*. Moreover, compared to BLP group, the gene expression level of *CYP2W1* was significantly upregulated in LPP group. However, there were two m6A peaks on mRNA of this gene, and the changes on m6A modification levels were opposite with one being upregulated and other downregulated. Thus, this fine tuning of m6A modifications of these two sites could be important for the post-transcription regulation of *CYP2W1* mRNA, which can regulate retinol metabolism in the ovary and promote the ovary development. Thus, we inferred that m6A might regulate ovary development through tightly controlling the expression of *CYP2W1* in retinol metabolism pathway.

Granulosa cells (GC), one of the follicular somatic cells, are critical in the follicle development. The proliferation of GC is a basic process which is required for normal follicular development (Wang et al., 2017). Moreover, impaired GC proliferation or GC apoptosis can lead to selective atresia of certain ovarian follicles. For instance, a previous review (Johnson, 2003) has indicated that the transition from a pre-hierarchal follicle to the preovulatory stage of the development is associated with dramatically increased resistance to apoptosis and increased cell proliferation in cultured hen GC. The preovulatory follicle viability is largely attributed to the acquired resistance of the GC layer to apoptosis. A recent study in pig ovarian somatic GC further demonstrated that p53 signaling pathway can inhibit GC cell cycle and then result in the follicle atresia (Li et al., 2021). The follicle atresia can be at any stage during development, and it has been identified as main reason for the reduction of egg production in chicken (He et al., 2022). Interestingly, functional analysis of DMPs and DEGs both demonstrated that pathways related to cell apoptosis such as “apoptosis” and “p53 signaling pathway” were enriched. *CASP18*, a member of caspase family, encoding caspase-18, was presented in this “apoptosis” pathway. The caspase family members play vital roles in the induction, transduction and amplification of intracellular apoptotic signals (Fan et al., 2005). In addition, compared to BLP group, m6A level and the gene expression level of *CASP18* were both significantly upregulated in LPP group, which indicated that cell apoptosis in the ovary was highly activated in the LPP group. However, in a similar study conducted by using the commercial chicken breed, the apoptosis related pathways were not found to be significantly enriched (Fan et al., 2019). The high level of m6A methylation of this gene might stabilize the mRNA and increase the levels of this mRNA, which leads to the apoptosis of GCs and further promote follicle atresia in LPP group. Therefore, we speculate that the follicles atresia due to the apoptosis of GC in LPP group could be the possible cellular mechanism underlying the low egg production of Wuhua yellow-feathered chicken in comparison to the commercial hen breeds. As a typical Chinese local chicken breed, Wuhua yellow-feathered chicken can exhibit higher broodiness compared to the commercial laying breeds which were under systematic and intensive selection for egg laying performance. Broodiness can effectively cause shrinking of the fallopian tubes and ovaries, thus inhibiting follicle development, promoting the appearance of atretic follicles and thereby reducing the granular layer and membrane cells in the follicles (Liu et al., 2018a). Overall, our results clearly indicate that the precise regulation of follicle development and follicle atresia in ovary of Wuhua yellow-feathered chicken was controlled by m6A methylation. It was also observed that the various apoptosis related pathways were the potential molecular mechanisms underlying the low egg production performance and the development of broodiness.

In this study, we generated a dynamic m6A transcriptional map of the Wuhua yellow-feathered chicken ovary before and after sexual maturation. Although some interesting and valuable results were found here, the limitation of current study is extremely clear. First, both the sample size at one stage and number of development stages were small. Only two separate development stages were taken into consideration here, which does not accurately depict the entire development process of chicken ovary and leaves out a wealth of information on the molecular regulatory mechanisms underlying this biological process. In addition, this study is purely omics-based and lacks multiple levels of validation tests. In the future, we aim to collect ovary samples in multiple development stages to analysis the dynamic changes of both m6A methylation level and expression level of candidate genes found in the current study. Besides, to clarify the potential impact of methylation on sexual maturity of Wuhua yellow-feathered chicken, histology tests and gene function validation on cell models were also required.

## Conclusion

We profiled the transcriptome-wide m6A methylation in Wuhua yellow-feathered chicken ovary before and after the sexual maturation stage. The precise expressional regulation of various genes related to the follicle development and follicle atresia controlled by m6A during the maturity can result in the poor reproductive performance in the Wuhua yellow-feathered chicken. The findings provide a solid foundation for further investigation of molecular mechanisms of ovary development and egg laying performance in Chinese indigenous chicken breeds. The pathways and corresponding candidate genes reported in this study could be useful for the molecular design breeding for improving egg production performance in Chinese local chicken breed, and it might also be beneficial for the genetic resource protection of the valuable avian species.

## Data Availability

The datasets presented in this study can be found in online repositories. The names of the repository/repositories and accession number(s) can be found below: https://www.ncbi.nlm.nih.gov/geo/, GSE239644.

## Supplementary material

SUPPLEMENTARY FIGURE S1 Pictures of the Wuhua yellow feathered chicken and ovary tissue sampling (before sexual maturation).

SUPPLEMENTARY FIGURE S2 Statistical analysis of sequencing data. **(A)** Mapping classification of each **(B)** m6A site classification of each **(C)** Peak length violin plot. **(D)** Rea ds distribution and density along the chicken genome.
