# Supplementary figures and images for: Transcriptome-wide m6A methylation profiling of Wuhua yellow-feathered chicken ovary revealed regulatory pathways underlying sexual maturation and low egg-laying performance
Source: Front Genet. 2023 Oct 20;14:1284554. doi: 10.3389/fgene.2023.1284554 (PMC10622773; doi:10.3389/fgene.2023.1284554)

**A**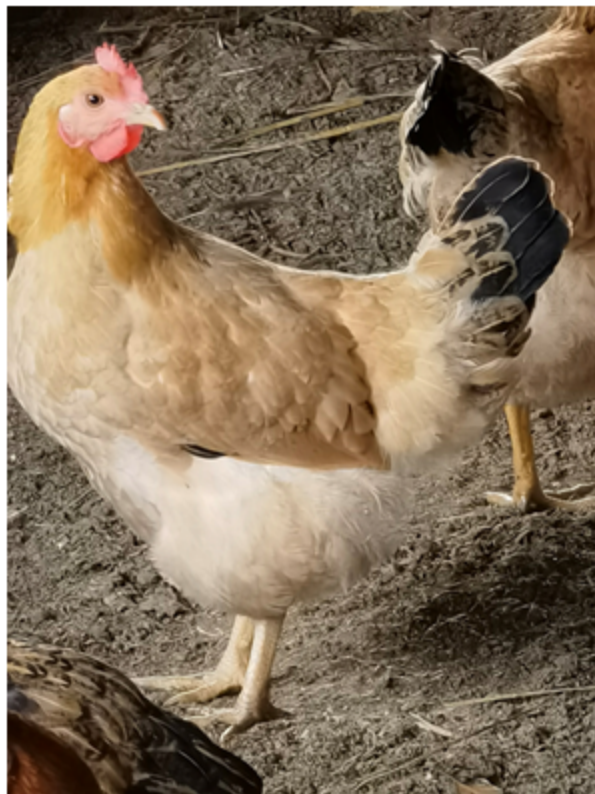**B**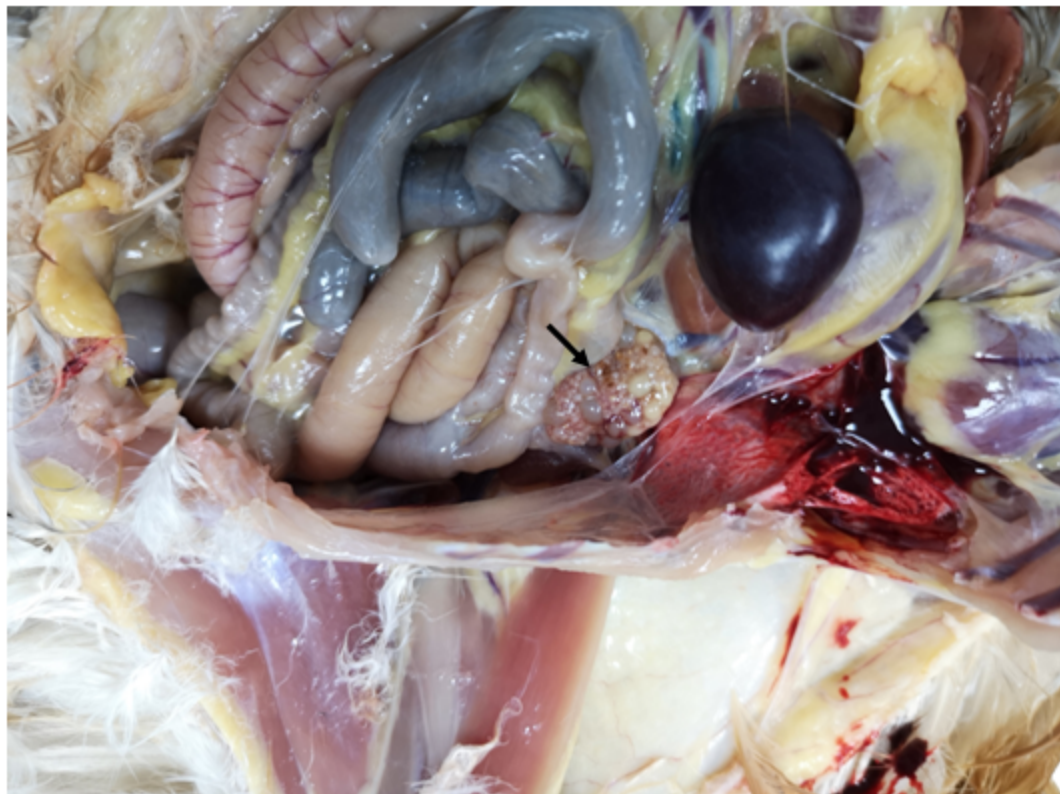

Supplement: Supplementary file 1 [file DataSheet1.ZIP › Supplementary Materials/Figure S1.pdf]

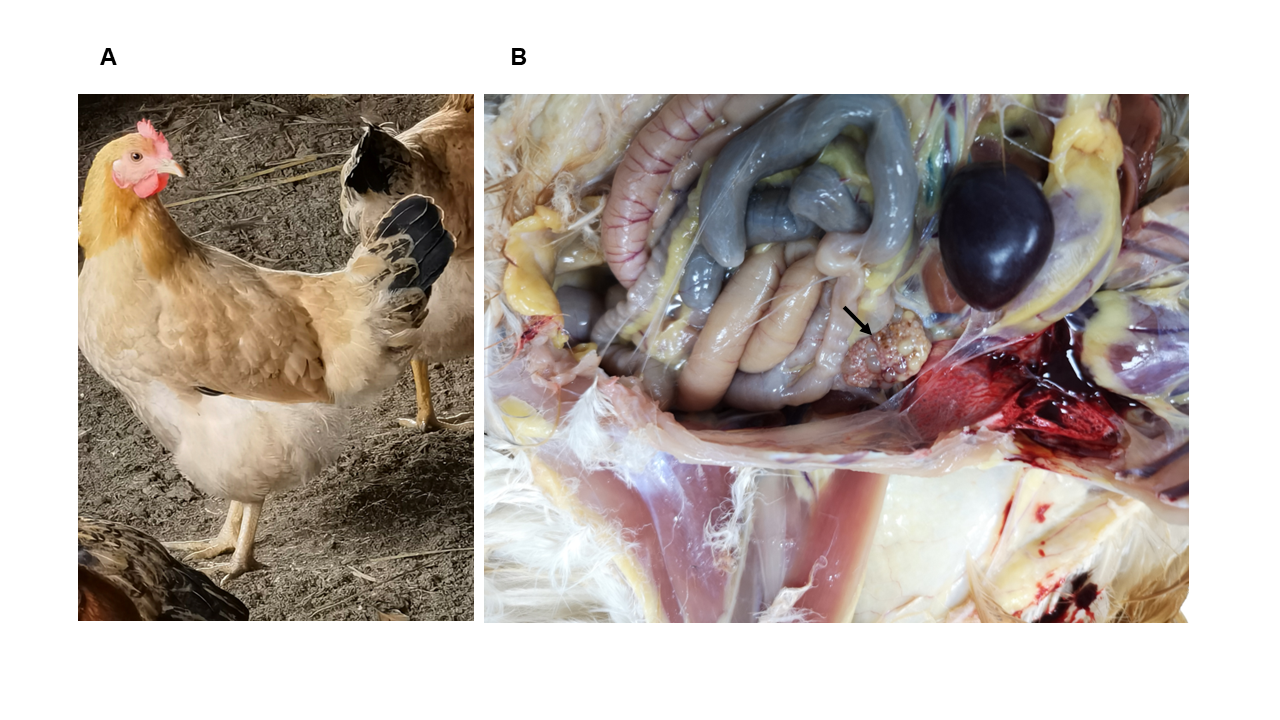

Supplement: Supplementary file 1 [file DataSheet1.ZIP › Supplementary Materials/Figure S1.tif]

A

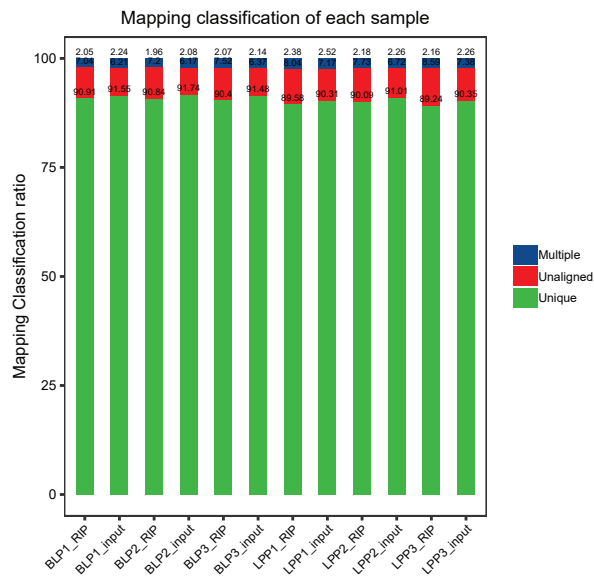

B

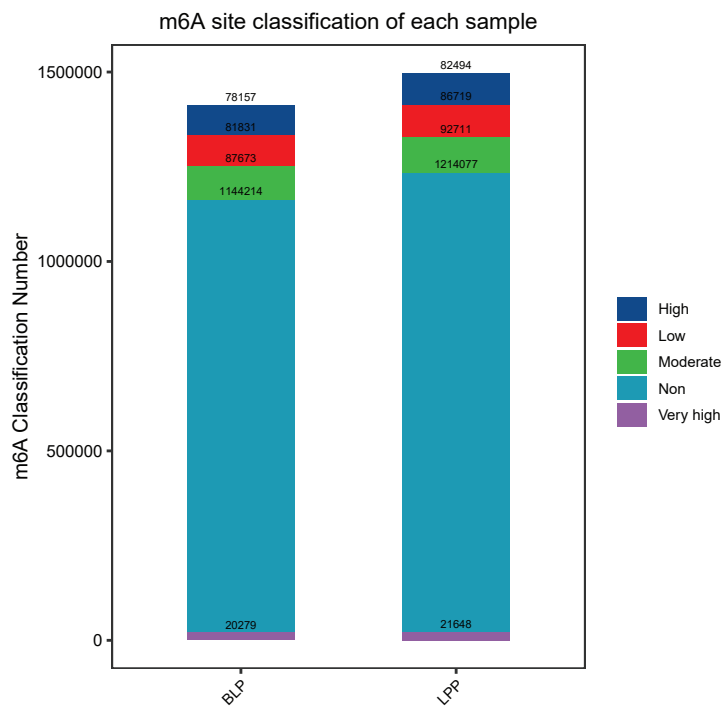

C

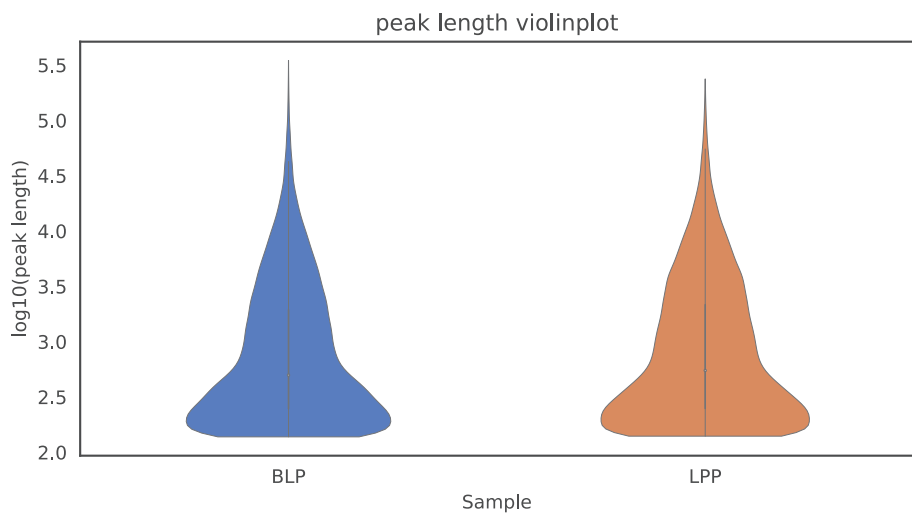

D

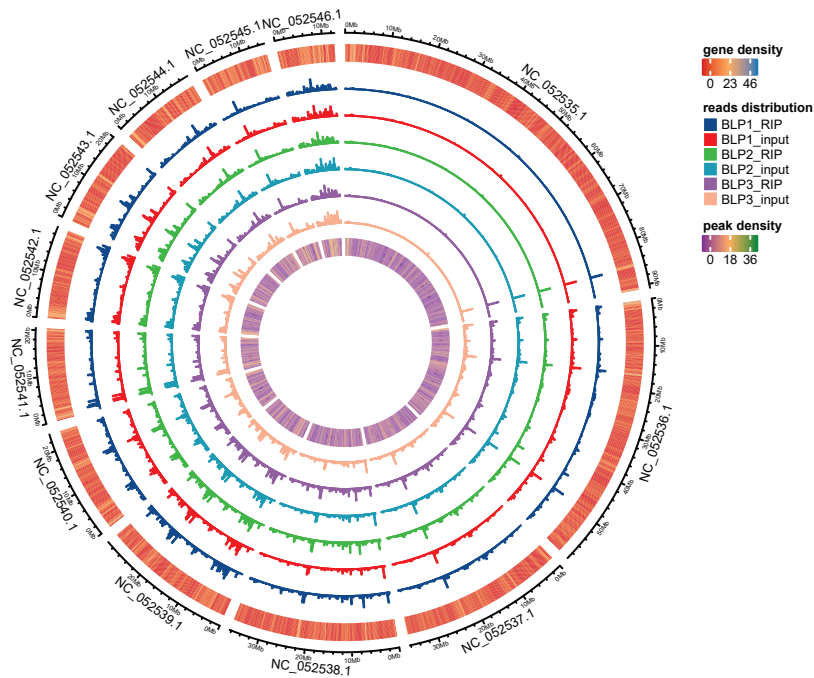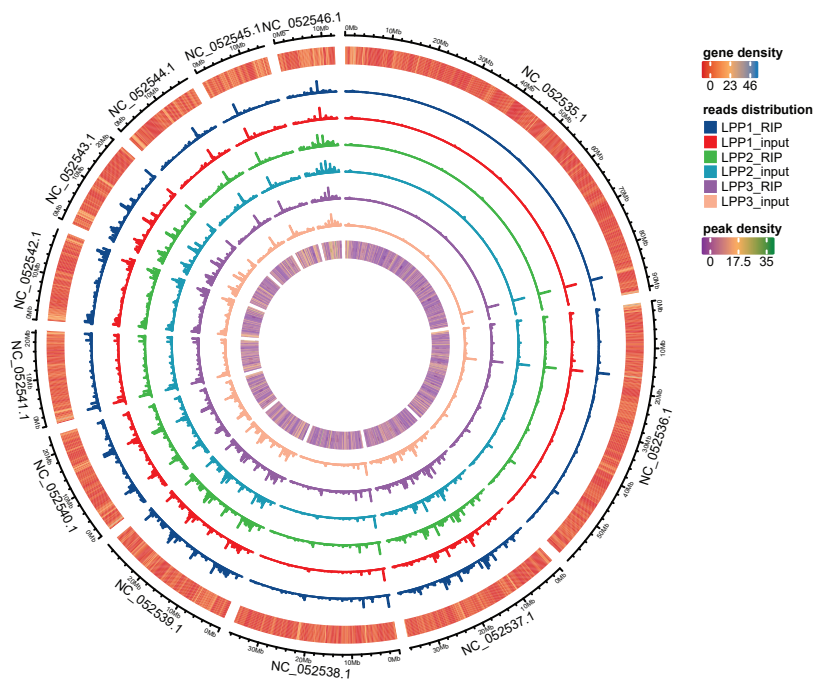

Supplement: Supplementary file 1 [file DataSheet1.ZIP › Supplementary Materials/Figure S2.pdf]
